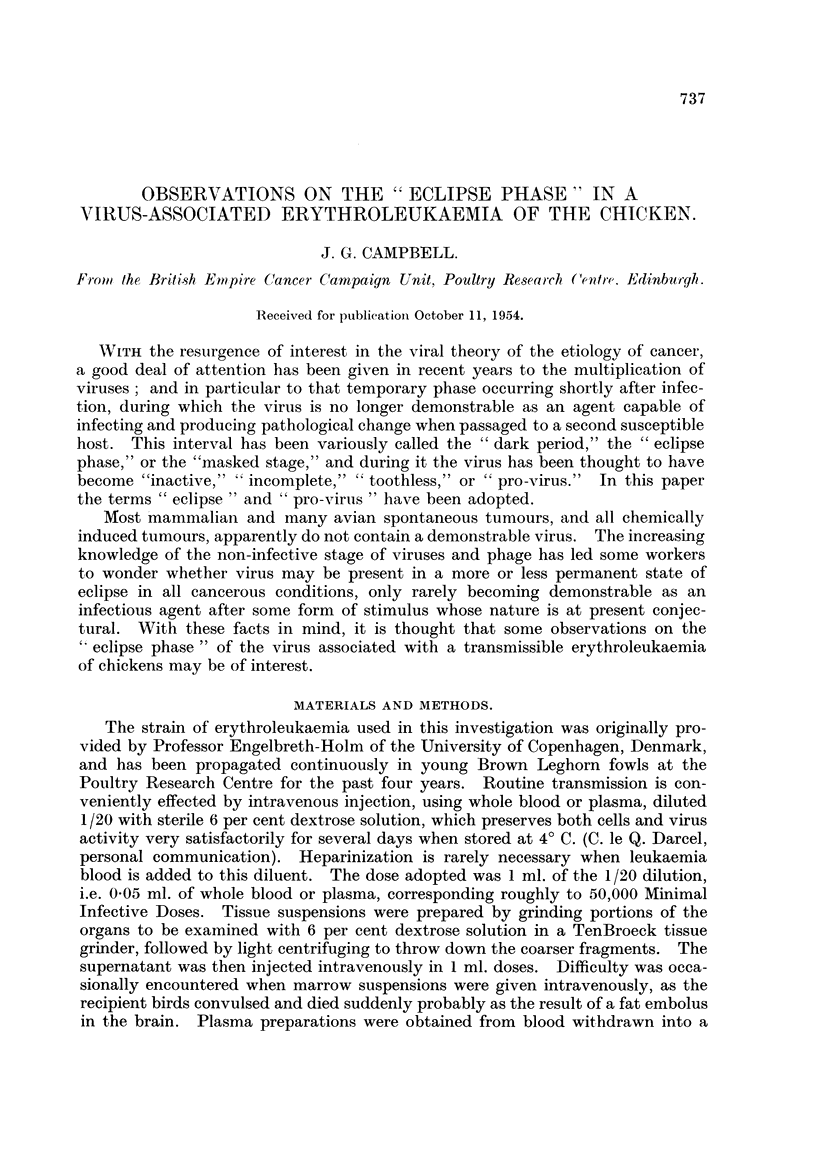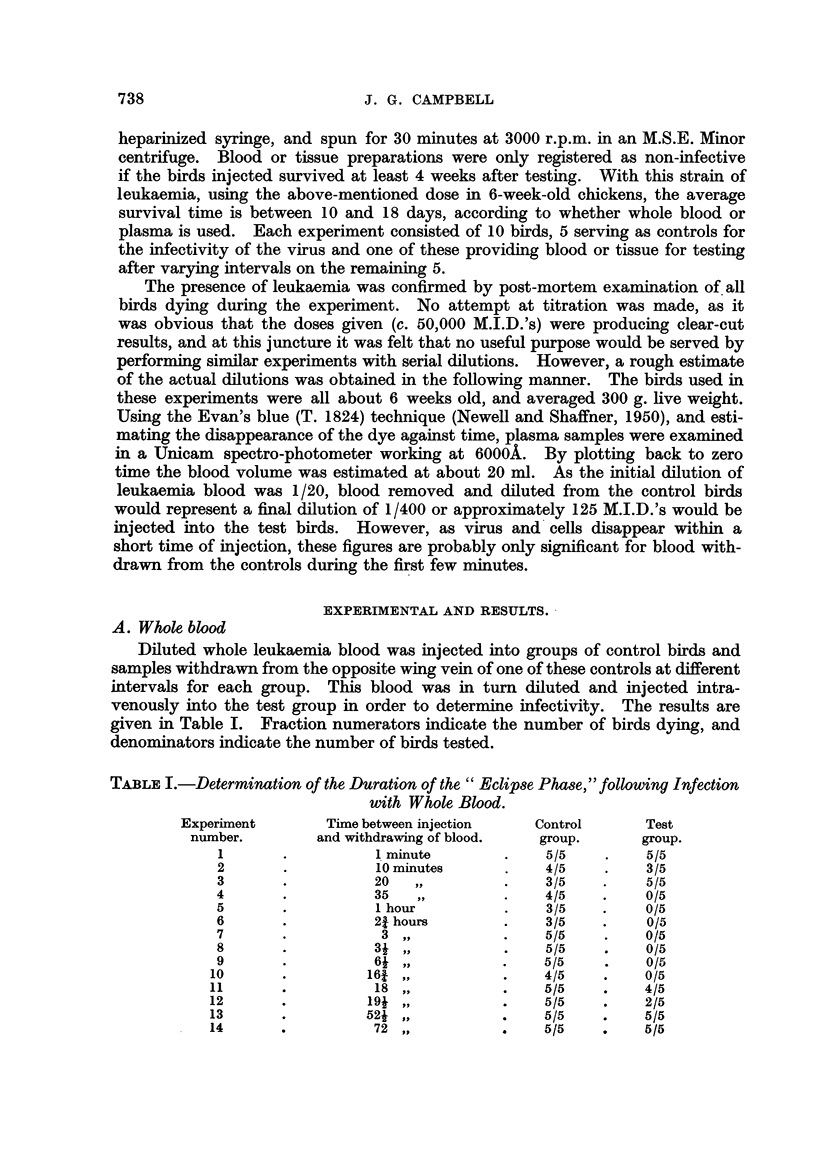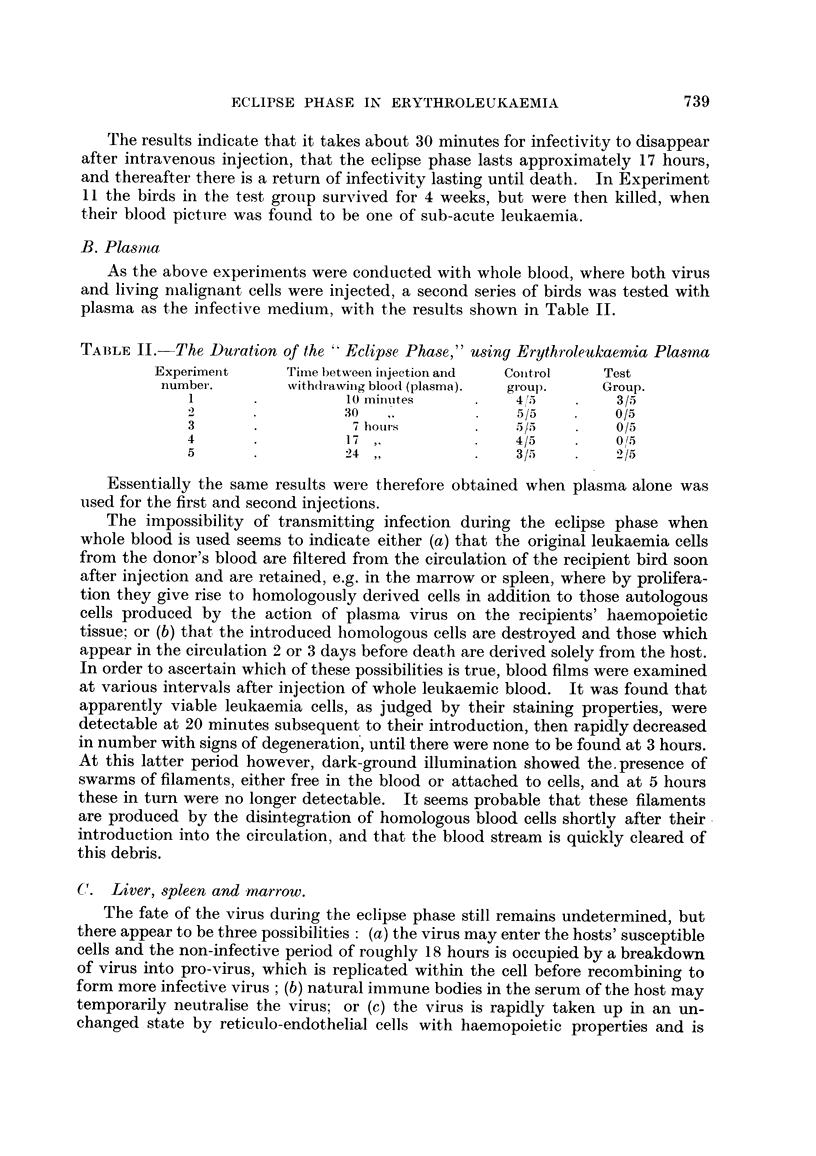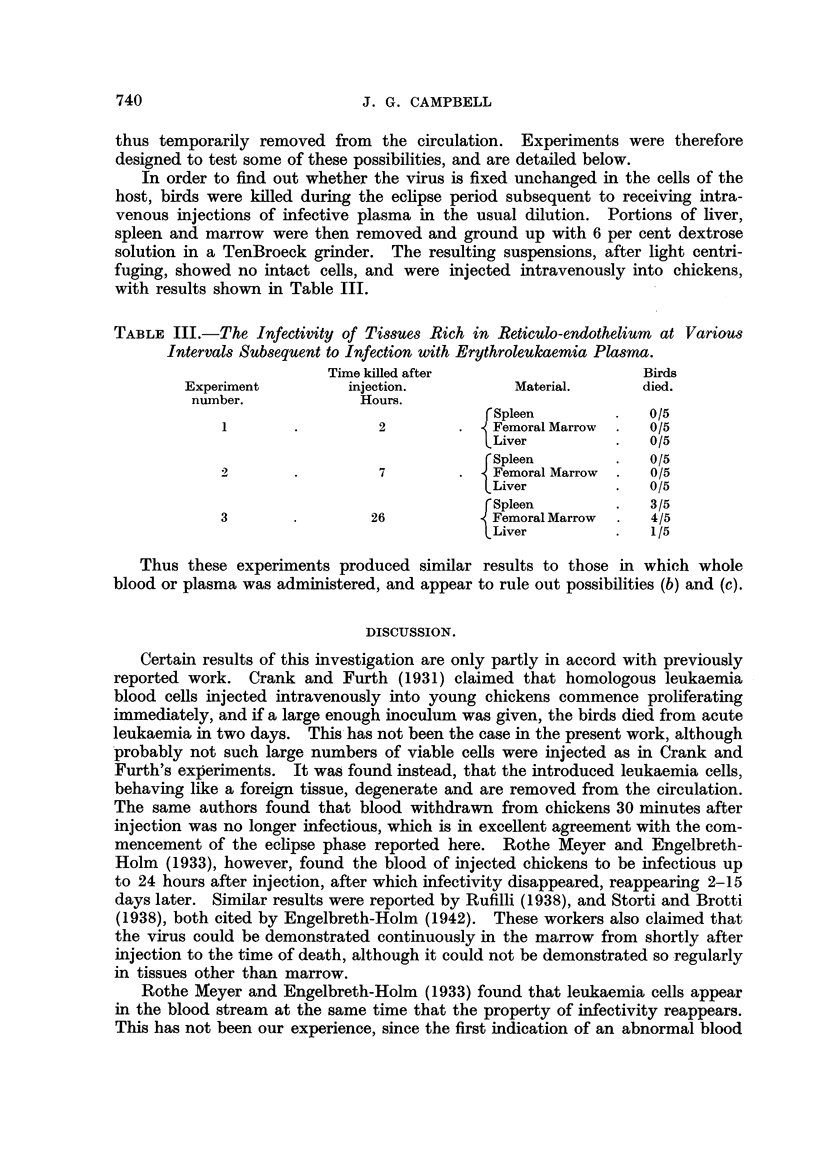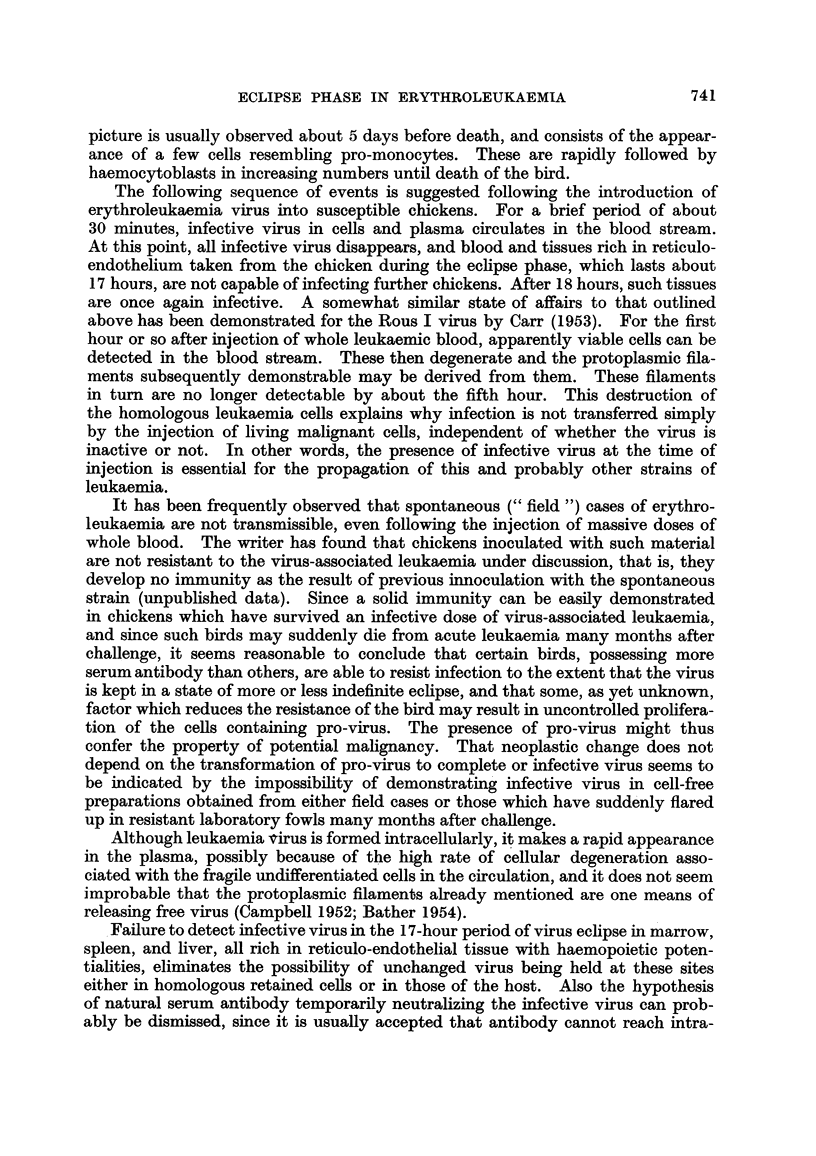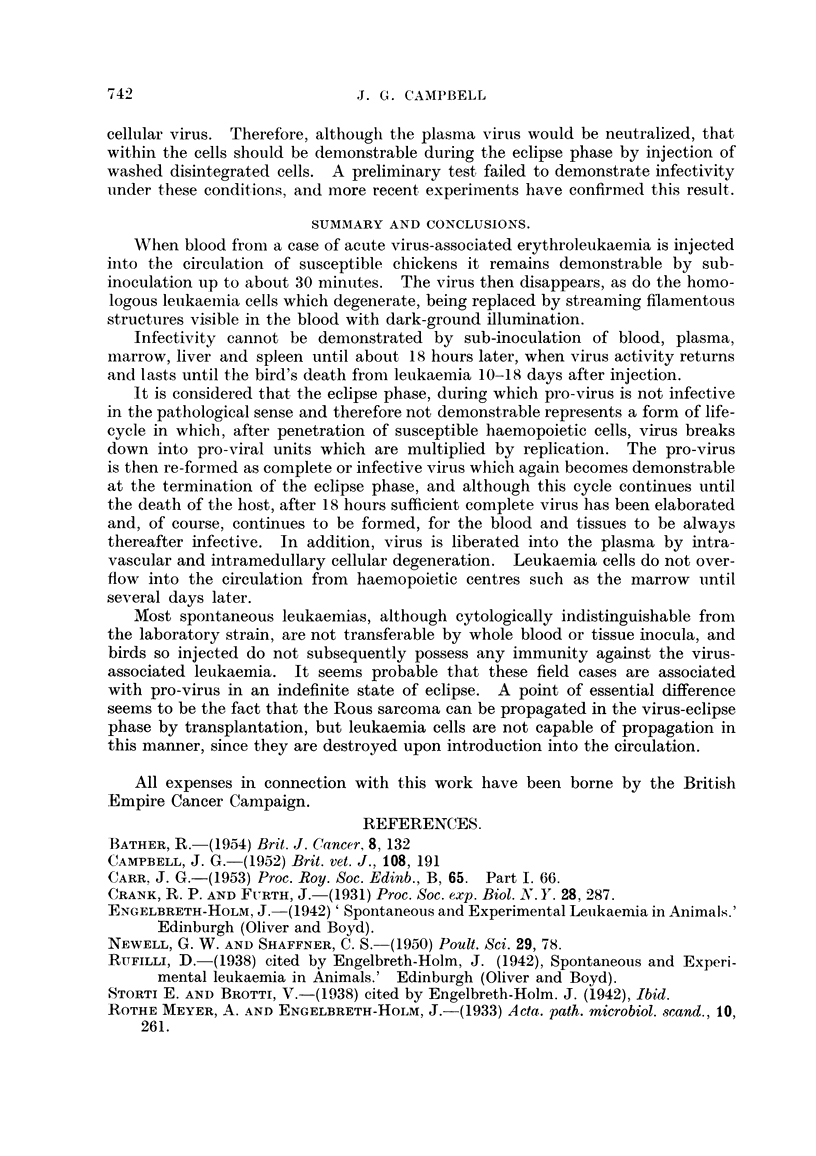# Observations on the “Eclipse Phase” in a Virus-associated Erythroleukaemia of the Chicken

**DOI:** 10.1038/bjc.1954.82

**Published:** 1954-12

**Authors:** J. G. Campbell


### OBSERVATIONS ON THE "ECLIPSE PHASE" IN A VIRUS-ASSOCIATED ERYTHROLEUKAEMIA OF THE CHICKEN.

J. G. CAMPBELL.

*From the British Empire Cancer Campaign Unit, Poultry Research Centre, Edinburgh.*

Received for publication October 11, 1954.

WITH the resurgence of interest in the viral theory of the etiology of cancer, a good deal of attention has been given in recent years to the multiplication of viruses ; and in particular to that temporary phase occurring shortly after infection, during which the virus is no longer demonstrable as an agent capable of infecting and producing pathological change when passaged to a second susceptible host. This interval has been variously called the "dark period," the "eclipse phase," or the "masked stage," and during it the virus has been thought to have become "inactive," "incomplete," "toothless," or "pro-virus." In this paper the terms "eclipse" and "pro-virus" have been adopted.

Most mammalian and many avian spontaneous tumours, and all chemically induced tumours, apparently do not contain a demonstrable virus. The increasing knowledge of the non-infective stage of viruses and phage has led some workers to wonder whether virus may be present in a more or less permanent state of eclipse in all cancerous conditions, only rarely becoming demonstrable as an infectious agent after some form of stimulus whose nature is at present conjectural. With these facts in mind, it is thought that some observations on the "eclipse phase" of the virus associated with a transmissible erythroleukaemia of chickens may be of interest.

### MATERIALS AND METHODS.

The strain of erythroleukaemia used in this investigation was originally provided by Professor Engelbreth-Holm of the University of Copenhagen, Denmark, and has been propagated continuously in young Brown Leghorn fowls at the Poultry Research Centre for the past four years. Routine transmission is conveniently effected by intravenous injection, using whole blood or plasma, diluted 1/20 with sterile 6 per cent dextrose solution, which preserves both cells and virus activity very satisfactorily for several days when stored at 4° C. (C. le Q. Darcel, personal communication). Heparinization is rarely necessary when leukaemia blood is added to this diluent. The dose adopted was 1 ml. of the 1/20 dilution, i.e. 0·05 ml. of whole blood or plasma, corresponding roughly to 50,000 Minimal Infective Doses. Tissue suspensions were prepared by grinding portions of the organs to be examined with 6 per cent dextrose solution in a TenBroeck tissue grinder, followed by light centrifuging to throw down the coarser fragments. The supernatant was then injected intravenously in 1 ml. doses. Difficulty was occasionally encountered when marrow suspensions were given intravenously, as the recipient birds convulsed and died suddenly probably as the result of a fat embolus in the brain. Plasma preparations were obtained from blood withdrawn into a heparinized syringe, and spun for 30 minutes at 3000 r.p.m. in an M.S.E. Minor centrifuge. Blood or tissue preparations were only registered as non-infective if the birds injected survived at least 4 weeks after testing. With this strain of leukaemia, using the above-mentioned dose in 6-week-old chickens, the average survival time is between 10 and 18 days, according to whether whole blood or plasma is used. Each experiment consisted of 10 birds, 5 serving as controls for the infectivity of the virus and one of these providing blood or tissue for testing after varying intervals on the remaining 5.

The presence of leukaemia was confirmed by post-mortem examination of all birds dying during the experiment. No attempt at titration was made, as it was obvious that the doses given (*c.* 50,000 M.I.D.'s) were producing clear-cut results, and at this juncture it was felt that no useful purpose would be served by performing similar experiments with serial dilutions. However, a rough estimate of the actual dilutions was obtained in the following manner. The birds used in these experiments were all about 6 weeks old, and averaged 300 g. live weight. Using the Evan's blue (T. 1824) technique (Newell and Shaffner, 1950), and estimating the disappearance of the dye against time, plasma samples were examined in a Unicam spectro-photometer working at 6000Å. By plotting back to zero time the blood volume was estimated at about 20 ml. As the initial dilution of leukaemia blood was 1/20, blood removed and diluted from the control birds would represent a final dilution of 1/400 or approximately 125 M.I.D.'s would be injected into the test birds. However, as virus and cells disappear within a short time of injection, these figures are probably only significant for blood withdrawn from the controls during the first few minutes.

### EXPERIMENTAL AND RESULTS.

### *A. Whole blood*

Diluted whole leukaemia blood was injected into groups of control birds and samples withdrawn from the opposite wing vein of one of these controls at different intervals for each group. This blood was in turn diluted and injected intravenously into the test group in order to determine infectivity. The results are given in Table I. Fraction numerators indicate the number of birds dying, and denominators indicate the number of birds tested.

TABLE I.—*Determination of the Duration of the "Eclipse Phase," following Infection with Whole Blood.*

| Experiment number. | Time between injection and withdrawing of blood. | Control group. | Test group. |
|---|---|---|---|
| 1 | 1 minute | 5/5 | 5/5 |
| 2 | 10 minutes | 4/5 | 3/5 |
| 3 | 20 ,, | 3/5 | 5/5 |
| 4 | 35 ,, | 4/5 | 0/5 |
| 5 | 1 hour | 3/5 | 0/5 |
| 6 | 2¾ hours | 3/5 | 0/5 |
| 7 | 3 ,, | 5/5 | 0/5 |
| 8 | 3½ ,, | 5/5 | 0/5 |
| 9 | 6½ ,, | 5/5 | 0/5 |
| 10 | 16¾ ,, | 4/5 | 0/5 |
| 11 | 18 ,, | 5/5 | 4/5 |
| 12 | 19½ ,, | 5/5 | 2/5 |
| 13 | 52½ ,, | 5/5 | 5/5 |
| 14 | 72 ,, | 5/5 | 5/5 |

The results indicate that it takes about 30 minutes for infectivity to disappear after intravenous injection, that the eclipse phase lasts approximately 17 hours, and thereafter there is a return of infectivity lasting until death. In Experiment 11 the birds in the test group survived for 4 weeks, but were then killed, when their blood picture was found to be one of sub-acute leukaemia.

*B. Plasma*

As the above experiments were conducted with whole blood, where both virus and living malignant cells were injected, a second series of birds was tested with plasma as the infective medium, with the results shown in Table II.

TABLE II.—*The Duration of the "Eclipse Phase," using Erythroleukaemia Plasma*

| Experiment number. | Time between injection and withdrawing blood (plasma). | Control group. | Test Group. |
|---|---|---|---|
| 1 | 10 minutes | 4/5 | 3/5 |
| 2 | 30 ,, | 5/5 | 0/5 |
| 3 | 7 hours | 5/5 | 0/5 |
| 4 | 17 ,, | 4/5 | 0/5 |
| 5 | 24 ,, | 3/5 | 2/5 |

Essentially the same results were therefore obtained when plasma alone was used for the first and second injections.

The impossibility of transmitting infection during the eclipse phase when whole blood is used seems to indicate either (*a*) that the original leukaemia cells from the donor's blood are filtered from the circulation of the recipient bird soon after injection and are retained, e.g. in the marrow or spleen, where by proliferation they give rise to homologously derived cells in addition to those autologous cells produced by the action of plasma virus on the recipients' haemopoietic tissue; or (*b*) that the introduced homologous cells are destroyed and those which appear in the circulation 2 or 3 days before death are derived solely from the host. In order to ascertain which of these possibilities is true, blood films were examined at various intervals after injection of whole leukaemic blood. It was found that apparently viable leukaemia cells, as judged by their staining properties, were detectable at 20 minutes subsequent to their introduction, then rapidly decreased in number with signs of degeneration, until there were none to be found at 3 hours. At this latter period however, dark-ground illumination showed the presence of swarms of filaments, either free in the blood or attached to cells, and at 5 hours these in turn were no longer detectable. It seems probable that these filaments are produced by the disintegration of homologous blood cells shortly after their introduction into the circulation, and that the blood stream is quickly cleared of this debris.

*C. Liver, spleen and marrow.*

The fate of the virus during the eclipse phase still remains undetermined, but there appear to be three possibilities : (*a*) the virus may enter the hosts' susceptible cells and the non-infective period of roughly 18 hours is occupied by a breakdown of virus into pro-virus, which is replicated within the cell before recombining to form more infective virus ; (*b*) natural immune bodies in the serum of the host may temporarily neutralise the virus; or (*c*) the virus is rapidly taken up in an unchanged state by reticulo-endothelial cells with haemopoietic properties and is thus temporarily removed from the circulation. Experiments were therefore designed to test some of these possibilities, and are detailed below.

In order to find out whether the virus is fixed unchanged in the cells of the host, birds were killed during the eclipse period subsequent to receiving intravenous injections of infective plasma in the usual dilution. Portions of liver, spleen and marrow were then removed and ground up with 6 per cent dextrose solution in a TenBroeck grinder. The resulting suspensions, after light centrifuging, showed no intact cells, and were injected intravenously into chickens, with results shown in Table III.

TABLE III.—*The Infectivity of Tissues Rich in Reticulo-endothelium at Various Intervals Subsequent to Infection with Erythroleukaemia Plasma.*

| Experiment number. | Time killed after injection. Hours. | Material. | Birds died. |
|---|---|---|---|
| | | Spleen | 0/5 |
| 1 | 2 | Femoral Marrow | 0/5 |
| | | Liver | 0/5 |
| | | Spleen | 0/5 |
| 2 | 7 | Femoral Marrow | 0/5 |
| | | Liver | 0/5 |
| | | Spleen | 3/5 |
| 3 | 26 | Femoral Marrow | 4/5 |
| | | Liver | 1/5 |

Thus these experiments produced similar results to those in which whole blood or plasma was administered, and appear to rule out possibilities (*b*) and (*c*).

### DISCUSSION.

Certain results of this investigation are only partly in accord with previously reported work. Crank and Furth (1931) claimed that homologous leukaemia blood cells injected intravenously into young chickens commence proliferating immediately, and if a large enough inoculum was given, the birds died from acute leukaemia in two days. This has not been the case in the present work, although probably not such large numbers of viable cells were injected as in Crank and Furth's experiments. It was found instead, that the introduced leukaemia cells, behaving like a foreign tissue, degenerate and are removed from the circulation. The same authors found that blood withdrawn from chickens 30 minutes after injection was no longer infectious, which is in excellent agreement with the commencement of the eclipse phase reported here. Rothe Meyer and Engelbreth-Holm (1933), however, found the blood of injected chickens to be infectious up to 24 hours after injection, after which infectivity disappeared, reappearing 2–15 days later. Similar results were reported by Rufilli (1938), and Storti and Brotti (1938), both cited by Engelbreth-Holm (1942). These workers also claimed that the virus could be demonstrated continuously in the marrow from shortly after injection to the time of death, although it could not be demonstrated so regularly in tissues other than marrow.

Rothe Meyer and Engelbreth-Holm (1933) found that leukaemia cells appear in the blood stream at the same time that the property of infectivity reappears. This has not been our experience, since the first indication of an abnormal blood picture is usually observed about 5 days before death, and consists of the appearance of a few cells resembling pro-monocytes. These are rapidly followed by haemocytoblasts in increasing numbers until death of the bird.

The following sequence of events is suggested following the introduction of erythroleukaemia virus into susceptible chickens. For a brief period of about 30 minutes, infective virus in cells and plasma circulates in the blood stream. At this point, all infective virus disappears, and blood and tissues rich in reticulo-endothelium taken from the chicken during the eclipse phase, which lasts about 17 hours, are not capable of infecting further chickens. After 18 hours, such tissues are once again infective. A somewhat similar state of affairs to that outlined above has been demonstrated for the Rous I virus by Carr (1953). For the first hour or so after injection of whole leukaemic blood, apparently viable cells can be detected in the blood stream. These then degenerate and the protoplasmic filaments subsequently demonstrable may be derived from them. These filaments in turn are no longer detectable by about the fifth hour. This destruction of the homologous leukaemia cells explains why infection is not transferred simply by the injection of living malignant cells, independent of whether the virus is inactive or not. In other words, the presence of infective virus at the time of injection is essential for the propagation of this and probably other strains of leukaemia.

It has been frequently observed that spontaneous (" field ") cases of erythroleukaemia are not transmissible, even following the injection of massive doses of whole blood. The writer has found that chickens inoculated with such material are not resistant to the virus-associated leukaemia under discussion, that is, they develop no immunity as the result of previous innoculation with the spontaneous strain (unpublished data). Since a solid immunity can be easily demonstrated in chickens which have survived an infective dose of virus-associated leukaemia, and since such birds may suddenly die from acute leukaemia many months after challenge, it seems reasonable to conclude that certain birds, possessing more serum antibody than others, are able to resist infection to the extent that the virus is kept in a state of more or less indefinite eclipse, and that some, as yet unknown, factor which reduces the resistance of the bird may result in uncontrolled proliferation of the cells containing pro-virus. The presence of pro-virus might thus confer the property of potential malignancy. That neoplastic change does not depend on the transformation of pro-virus to complete or infective virus seems to be indicated by the impossibility of demonstrating infective virus in cell-free preparations obtained from either field cases or those which have suddenly flared up in resistant laboratory fowls many months after challenge.

Although leukaemia virus is formed intracellularly, it makes a rapid appearance in the plasma, possibly because of the high rate of cellular degeneration associated with the fragile undifferentiated cells in the circulation, and it does not seem improbable that the protoplasmic filaments already mentioned are one means of releasing free virus (Campbell 1952; Bather 1954).

Failure to detect infective virus in the 17-hour period of virus eclipse in marrow, spleen, and liver, all rich in reticulo-endothelial tissue with haemopoietic potentialities, eliminates the possibility of unchanged virus being held at these sites either in homologous retained cells or in those of the host. Also the hypothesis of natural serum antibody temporarily neutralizing the infective virus can probably be dismissed, since it is usually accepted that antibody cannot reach intracellular virus. Therefore, although the plasma virus would be neutralized, that within the cells should be demonstrable during the eclipse phase by injection of washed disintegrated cells. A preliminary test failed to demonstrate infectivity under these conditions, and more recent experiments have confirmed this result.

### SUMMARY AND CONCLUSIONS.

When blood from a case of acute virus-associated erythroleukaemia is injected into the circulation of susceptible chickens it remains demonstrable by sub-inoculation up to about 30 minutes. The virus then disappears, as do the homologous leukaemia cells which degenerate, being replaced by streaming filamentous structures visible in the blood with dark-ground illumination.

Infectivity cannot be demonstrated by sub-inoculation of blood, plasma, marrow, liver and spleen until about 18 hours later, when virus activity returns and lasts until the bird's death from leukaemia 10–18 days after injection.

It is considered that the eclipse phase, during which pro-virus is not infective in the pathological sense and therefore not demonstrable represents a form of life-cycle in which, after penetration of susceptible haemopoietic cells, virus breaks down into pro-viral units which are multiplied by replication. The pro-virus is then re-formed as complete or infective virus which again becomes demonstrable at the termination of the eclipse phase, and although this cycle continues until the death of the host, after 18 hours sufficient complete virus has been elaborated and, of course, continues to be formed, for the blood and tissues to be always thereafter infective. In addition, virus is liberated into the plasma by intravascular and intramedullary cellular degeneration. Leukaemia cells do not overflow into the circulation from haemopoietic centres such as the marrow until several days later.

Most spontaneous leukaemias, although cytologically indistinguishable from the laboratory strain, are not transferable by whole blood or tissue inocula, and birds so injected do not subsequently possess any immunity against the virus-associated leukaemia. It seems probable that these field cases are associated with pro-virus in an indefinite state of eclipse. A point of essential difference seems to be the fact that the Rous sarcoma can be propagated in the virus-eclipse phase by transplantation, but leukaemia cells are not capable of propagation in this manner, since they are destroyed upon introduction into the circulation.

All expenses in connection with this work have been borne by the British Empire Cancer Campaign.

### REFERENCES.

Bather, R.—(1954) *Brit. J. Cancer*, **8**, 132

Campbell, J. G.—(1952) *Brit. vet. J.*, **108**, 191

Carr, J. G.—(1953) *Proc. Roy. Soc. Edinb.*, B, **65**. Part I. 66.

Crank, R. P. and Furth, J.—(1931) *Proc. Soc. exp. Biol. N.Y.* **28**, 287.

Engelbreth-Holm, J.—(1942) ' Spontaneous and Experimental Leukaemia in Animals.' Edinburgh (Oliver and Boyd).

Newell, G. W. and Shaffner, C. S.—(1950) *Poult. Sci.* **29**, 78.

Rufilli, D.—(1938) cited by Engelbreth-Holm, J. (1942), Spontaneous and Experimental leukaemia in Animals.' Edinburgh (Oliver and Boyd).

Storti E. and Brotti, V.—(1938) cited by Engelbreth-Holm. J. (1942), *Ibid.*

Rothe Meyer, A. and Engelbreth-Holm, J.—(1933) *Acta. path. microbiol. scand.*, **10**, 261.